# Exosomes promote pre-metastatic niche formation in ovarian cancer

**DOI:** 10.1186/s12943-019-1049-4

**Published:** 2019-08-13

**Authors:** Wenlong Feng, Dylan C. Dean, Francis J. Hornicek, Huirong Shi, Zhenfeng Duan

**Affiliations:** 1 Department of Obstetrics and Gynecology, The First Affiliated Hospital of Zhengzhou University, 1 Jianshe East Road, Zhengzhou, 450052 Henan China; 2 Department of Orthopaedic Surgery, David Geffen School of Medicine at UCLA, 615 Charles, E. Young. Dr. South, Los Angeles, CA 90095 USA

**Keywords:** Ovarian cancer, Exosome, Pre-metastatic niche, Metastasis

## Abstract

Ovarian cancer is one of the most common gynecological malignancies. Upon initial diagnosis, the majority of patients present with widespread metastatic growth within the peritoneal cavity. This metastatic growth occurs in stages, with the formation of a pre-metastatic niche occurring prior to macroscopic tumor cell invasion. Exosomes released by the primary ovarian tumor are small extracellular vesicles which prepare the distant tumor microenvironment for accelerated metastatic invasion. They regulate intercellular communication between tumor cells and normal stroma, cancer-associated fibroblasts, and local immune cells within the tumor microenvironment. In this review, we highlight the emerging roles of ovarian cancer exosomes as coordinators of pre-metastatic niche formation, biomarkers amenable to liquid biopsy, and targets of chemotherapy.

## Highlights

- Ovarian cancer is the deadliest gynecological malignancy, largely stemming from peritoneal metastasis. Formation of the pre-metastatic niche supports subsequent metastatic lesions.
- Ovarian cancer derived exosomes induce pre-metastatic niche formation via immunosuppression, angiogenesis, stromal cell remodeling, and oncogenic reprogramming.
- Ovarian cancer derived exosomes are promising biomarkers and therapeutic targets.

## Background

Ovarian cancer is the most lethal gynecological malignancy, accounting for 2.5% of all female cancers and 5% of female cancer-related deaths [1]. In the United States, there were approximately 22,240 new cases of ovarian cancer diagnosed and 14,070 deaths in 2018 [2]. Ovarian cancer patients experience high mortality rates due to commonly being diagnosed during the late stage (III or IV) with bowel obstruction and systemic involvement [3]. The 5-year survival rate of ovarian cancer among these late-stage III or IV cases is less than 29%, as compared to 70% in early stage I cases [4]. There is, therefore, an urgent need to characterize the mechanisms of ovarian cancer metastasis and associated biomarkers in order to earlier diagnose and treat ovarian cancer patients within the stage I period.

**Fig. 1:**
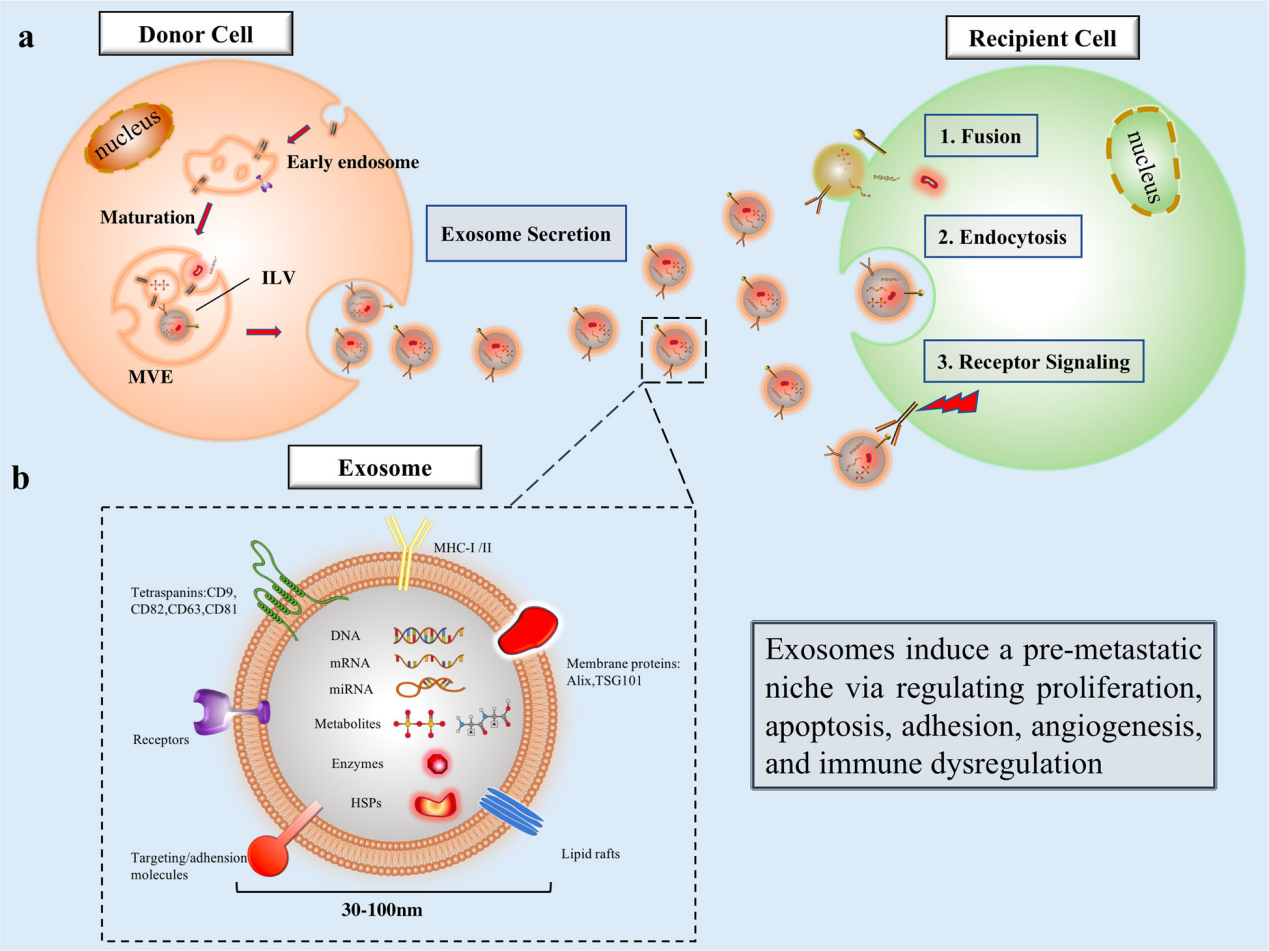
**a**. The biogenesis of exosomes and the mechanisms involved in intercellular communication. The exosome is an intraluminal vesicle (ILV) formed by the inward budding of the endosomal membrane during the maturation of the multivesicular endosomes (MVEs) and then secreted after fusing with the cell surface. Uptake by recipient cells occurs via a three step processes: 1. membrane fusion with target cells; 2. endocytosis; 3. activation of surface receptors and signaling. **b**. Schematic diagram of the exosome. The exosome is a disk-shaped membranous vesicle with a diameter 30–100 nm, and carries a parental cell cargo including lipids, metabolites, proteins, nucleic acids (DNA fragments, mRNA, miRNA, etc.)

Recent works have shown the pre-metastatic niche in ovarian cancer to be a prevalent precondition of metastasis [5]. The pre-metastatic niche is a preformed microenvironment made possible by exosomes secreted by the primary tumor site prior to widespread metastasis [6–8]. These exosomes optimize the environment for ovarian cancer colonization, outgrowth, and metastasis [9, 10]. This environment is mediated through immune suppression and evasion, angiogenesis, cancer-associated fibroblasts (CAF), and tumor macrophages that remodel the local stroma. Various studies have shown exosomes to play critical roles in tumorigenesis, growth, apoptosis, immune response, and chemotherapeutic resistance in cancer [11–15] (Fig. 1a). However, it was not until very recently that their roles in pre-metastatic niche establishment have been appreciated [15, 16]. Despite their diameter of 30–100 nm, these disk-shaped membranous vesicles contain unique signatures of proteins, lipids, DNA, and RNA making them powerful tumorigenic factors and diagnostic biomarkers [17]. In ovarian cancer, exosomes are detectable in both ascites and blood, making them amenable to less invasive diagnostics and a potential target of earlier targeted therapy [18–20]. In this review, we summarize the functions of exosomes in ovarian cancer metastasis with respect to pre-metastatic niche formation.

### Exosome biogenesis

The nature and abundance of exosomes are dictated by their donor cell of origin — whether it be healthy tissue or tumor [21]. Exosome biogenesis involves a set sequence of cellular events which eventually render a carcinogenic or physiologic subtype. The donor cells first internalize extracellular ligands and products to form early endosomes. Nascent exosomes then arise as intraluminal vesicles (ILVs) within the lumen of these endosomes, eventually maturing into multivesicular endosomes (MVEs) via selective inclusion of proteins, nucleic acids, and lipids. Whereas some MVEs fuse and degrade within lysosomes to provide the necessary energy and materials for cell formation, others are released into the extracellular environment through Golgi recirculation or direct cell secretion [22]. The endosomal sorting complex required for transport (ESCRT) is a primary driver of membrane shaping and scission, and the first accepted mechanism of MVE and ILV formation [23]. The final exosomal cargo drives its eventual function, and is achieved by a highly selective series of regulatory stages. It involves endoprotein sorting and precise ESCT cleavage of ubiquitinated proteins into ILVs via ESCT-0, −I, −II, and -III as well as VPS4, VTA1, ALIX/PDCD6IP, and TSG101. Lipids such as ceramide, cholesterol, and the four-transmembrane protein superfamily including CD9, CD63, and CD81 also mediate exosome protein sorting. At the nucleic acid level, exosomal mRNAs are sorted into MVEs according to the Z-zipper structure of their 3′-UTR as well as through RNA-induced silencing complexes (RISCs) [24, 25]. An ARF6–Exportin-5 axis also delivers pre-miRNA cargo into tumor microvesicles [26]. These regulatory methods demonstrate the mechanism by which ovarian cancer exosomes obtain their distinct molecular signature from surrounding healthy tissue.

### Ovarian cancer-exosomes

Ovarian cancer is distinguished from other human tumors by its preference for peritoneal cavity invasion through the ascites, making it especially adept at involving various viscera within the compartment. From the early stages on, the ascites contains detached tumor cells, various immune cells, mesothelial cells, and tumor associated exosomes. These exosomes can be isolated from the ascites [27, 28] and serum [29, 30] of patients with ovarian cancer. Importantly, these exosomes contain unique protein signatures specific to ovarian cancer, including membrane proteins (Alix, TSG 101), small GTPases (Rab proteins), annexin proteins, tetraspanins (CD9, CD82, CD63 and CD81), heat shock proteins (Hsp90, Hsc70), antigens (MHC I and II), Nanog and enzymes (phosphate isomerase, peroxiredoxin, aldehyde reductase, fatty acid synthase) (Fig. 1b). In addition to revealing an underlying malignancy, the exosomal protein cargo functions to enhance the progression of metastasis of ovarian tumors (Table 1). For example, Nanog is a transcription regulator involved in tumor cell proliferation and self-renewal of cancer stem cells [31]. Nanog expression is significantly greater in exosomes sampled from the ascites of high-grade serous ovarian cancer compared to benign peritoneal fluid [32]. Nanog knockout studies have shown decreased migration and invasion of ovarian cancer cells [33].

**Table 1:** Summary of ovarian cancer exosomal proteins involved with metastasis

Exosomal Proteins	Recipient cells	Role/Mechanism	Reference
Proteomic analysis: 2230 proteins were identified	N/A	Tumorigenesis and metastasis	[34]
ATF2, MTA1, ROCK1/2	HUVECs	Angiogenesis	[35]
GNA12, EPHA2 and COIA1	MSCs and ECs	Promote MSC and EC migration for metastasis	[36]
CD44	HPMCs	Tumor cell invasion.	[37]
RNA-binding protein LIN28	HEK293 cells	Increase HEK293 cell invasion and migration	[38]
Nanog	N/A	Tumor cell proliferation and invasion	[32, 33]

*Abbreviations*: *ATF2* Activating transcription factor 2, *MTA1* Metastasis-associated protein 1, *ROCK* Rho-Associated, Coiled-Coil Containing Protein Kinase, *HUVECs* Human umbilical vein endothelial cells, *GNA12* Guanine nucleotide-binding protein subunit alpha-12, *EPHA2* ephrin type-A receptor 2, *COIA1* Collagen alpha-1 (XVIII) chain, *MSCs* Mesenchymal stem cells, *ECs* endothelial cells, *HPMCs* Human peritoneal mesothelial cells, *LIN28* Lin-28 homolog A, *HEK293* human embryonic kidney

Tumorigenic microRNAs (miRNA) have also been identified within exosomes, where they are encapsulated and protected from nuclease degradation. Functionally, these intra-exosomal miRNAs regulate gene expression of target cells both locally and systemically. Once shed, the ovarian cancer derived exosomes horizontally transport their miRNAs to tumor-associated macrophages (TAMs), mesothelial cells, and tumor cells themselves. They provide instructions for pre-metastatic niche formation and metastasis in the nascent stages of malignancy (Table 2).

**Table 2:** Summary of ovarian cancer exosomal miRNAs involved with metastasis

Exosomal miRNAs	Recipient cells	Role/Mechanism	Reference
miR-940, miR-222-3p,miR-21–3p, miR-125 b-5p, miR-181 d-5p	TAMs	M2 phenotype polarization, EOC proliferation and migration	[39–41]
miRNA 21 and 29a	ES2 OC cellsLP9 mesothelial cells	Mesothelial cell clearance	[42]
miR-99a-5p	HPMC	Cell invasion through fibronectin and vitronectin upregulation	[43]
MMP1 mRNAs	MeT-5A and HPMC	Destruction of peritoneal mesothelium barrier	[44]
let-7a-f and miR-200a-c	N/A	Correlates with ovarian cancer invasiveness	[45]

*Abbreviations: TAMs* Tumor-associated macrophages, *HPMC* Human peritoneal mesothelial cells, *OC* ovarian cancer, *EOC* Epithelial ovarian cancer, *MMP* Matrix metallopeptidase

### Exosomes cause immunosuppression within the pre-metastatic niche

**Fig. 2:**
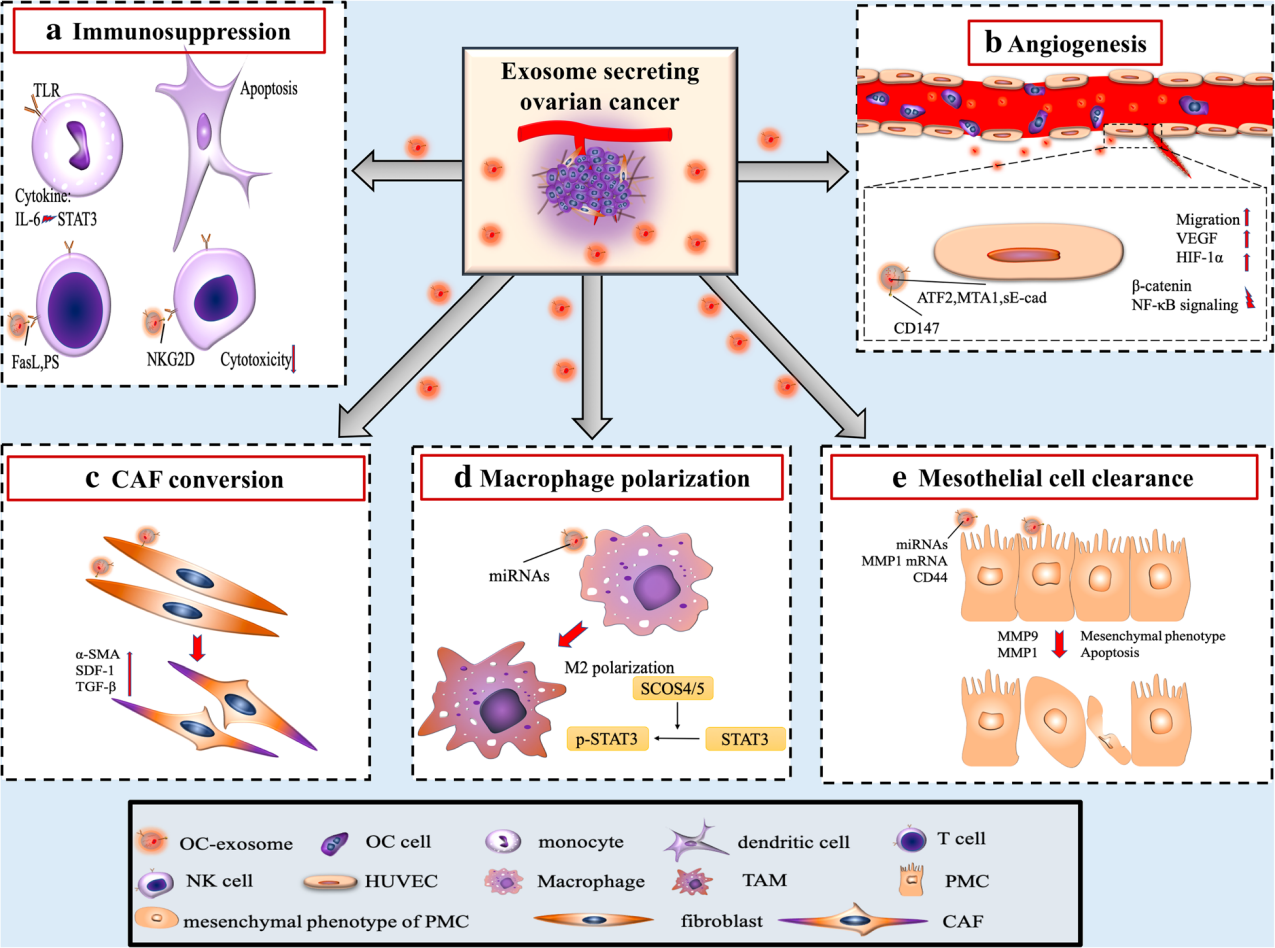
Impact of ovarian cancer-exosomes on target cells during pre-metastatic niche formation. **a**. Ovarian cancer-exosomes induce apoptosis of dendritic cells, increase secretion of IL-6, and inhibit function of T cells and NK cells. **b**. Ovarian cancer-exosomes with their cargo, such as ATF2, MTA1, sE-cad, and CD147, which acts on human vein endothelial cells, inducing angiogenesis and vascular permeability. **c**. Ovarian cancer-exosomes convert fibroblasts to CAFs. **d**. Ovarian cancer-exosomes deliver miRNAs to macrophages and elicit M2 macrophage polarization by regulating the suppressor of cytokine signaling (SOCS)4/5/ STAT3 pathway in macrophages. **e**. Phenotypic conversion and apoptosis of PMCs, induced by ovarian cancer-exosomes, clears the mesothelial cell barrier of the peritoneum and omentum. *OC* ovarian cancer, *NK* natural killer, *ATF2* Activating transcription factor 2, *MTA1* Metastasis-associated protein 1, *sE-cad* soluble E-cadherin, *SOCS* Suppress cytokine signaling, *STAT3* Signal transducer and activator of transcription 3, *PMC* Peritoneal mesothelial cell, *CAF* cancer-associated fibroblast

The immune system is a significant barrier to metastasis. For ovarian cancer to thrive in its new environment, it is therefore principally important for the premetastatic niche to protect the metastatic cells from being apoptosed upon entering the metastatic site. Exosomes isolated from the ascites of ovarian cancer can induce a rapid and reversible T cell arrest [17]. One recent study found GD3, a ganglioside expressed on the surface of exosomes isolated from ascites, to arrest T cells via acting on their T-cell receptor (TCR) [46]. Ovarian cancer associated exosomes can also induce the production of IL-6 within monocytes through toll-like receptor (TLR) activation. IL-6 then activates the signal transducer and activator of transcription 3 (STAT3) pathway in immune cells, stromal cells, and tumor cells, which supports overall immune escape of cancer cells [47] (Fig. 2a).

With the aim of revealing the direct effect of exosomes on immune cells, various works have cocultured exosomes from the ascites of ovarian cancer patients with dendritic cells, hematopoietic stem cells, and peripheral blood lymphocytes. One such study showed exosomes to cause immunosuppression by inducing apoptosis of hematopoietic stem cells, dendritic cells, and peripheral blood lymphocytes [27] (Fig. 2a). In another study, normal peripheral blood lymphocytes were cocultured with exosomes from ovarian cancer ascites or peritoneal washings of patients with benign ovarian cysts with follow up gene expression analysis of the lymphocytes. They found overexpression of 26 immunosuppressive genes in the malignant ascites cocultured group compared to the benign ovarian cyst group. These findings support the role of exosomes in dampening peripheral immunity through direct interaction with leukocytes, allowing for unmitigated tumor invasion [48].

Both the adaptive immune response, including CD4+ and CD8+ T cells, as well as innate immune cells such as natural killer (NK) cells and macrophages, are strongly recruited to the metastatic site. These immune cells are potent physiologic defenses against tumor progression [49]. A growing body of evidence suggests exosomes derived from ovarian cancer cells can silence these immune cells in the tumor microenvironment and are critical in pre-metastatic niche formation [50–52]. In addition to reprogramming the immune cell gene profile, ovarian cancer cells release Fas ligand (FasL)-carrying exosomes which downregulate the expression of the surface T-cell receptor/CD3- zeta (ζ) and promote T-cell apoptosis [53, 54]. At the cellular level, lysophosphatidic acid elevates the expression of FasL on the surface of ovarian cancer cells, thus prompting the release of FasL-carrying exosomes [55]. In addition, ovarian cancer exosomes carry phosphatidylserine, which inhibits T-cell activation by blocking intracellular signaling cascades [56]. NK cells are prominent innate immune effector cells that recognize and kill tumor cells directly. The NKG2D receptor-ligand is a crucial activating cytotoxic receptor of NK cells and a target of ovarian cancer exosome downregulation. Inhibition of NKG2D circumvents NK cell cytotoxicity, allowing for immune evasion of tumor cells and a robust pre-metastatic niche [57] (Table 3) (Fig. 2a).

**Table 3:** Roles of ovarian cancer exosomes in pre-metastatic niche formation

Exosome Function	Origin of exosomes	Recipient cells	Effect	Potential targets	Ref.
Immunosuppression	Ascites of OC patients	T cells	Arrest T cells	GD3, TCR	[46]
	Ascites of OC patients	Monocytes	Cytokine production	TLR signaling pathways	[47]
	Ascites of OC patients	PBMCs and DCs	Apoptosis	FasL	[27]
	Ascites of OC patients	T cells	Apoptosis	FasL	[54]
	OC cells	T cells	Counterattack against activated T cells	LPA and FasL	[55]
	Ovarian tumor tissues	T cells	T cell inhibition	Depletion or blockade of PS	[56]
	EOC cells and ascites	NK cells	Cytotoxicity inhibition	NKG2D	[57]
Angiogenesis	OC cells	HUVECs	Affect VEGF or HIF-1α	ATF2 and MTA1	[58]
	OC cells	HUVECs	CD147 stimulates VEGF expression	CD147	[59]
	OC cells	HUVECs	Activate β-catenin and NF-κB signaling	sE-cad	[60]
Stroma remodeling	OC cells	ADSCs	Transition ADSCs to CAFs	α-SMA, SDF-1, TGF-β SMAD2 and PI3K/AKT pathway	[67]
	OC cells	Macrophages	Macrophage M2 polarization	miR-21–3p, miR-125b-5p, and miR-181d-5p SOCS4/5/ STAT3 pathway	[41]

*Abbreviations*: *OC* ovarian cancer, *GD 3* ganglioside 3, *TCR* T-cell receptor, *TLR* Toll-like receptor, *PBMCs* peripheral blood lymphocytes, *DCs* dendritic cells, *FasL* Fas ligand, *LPA* Lysophosphatidic acid, *PS* phosphatidylserine, *EOC* Epithelial ovarian cancer, *NK* natural killer, *HIF* Hypoxia-inducible factor, *HUVECs* Human umbilical vein endothelial cells, *sE-cad* soluble E-cadherin, *ADSCs* adipose tissue-derived mesenchymal stem cells, *α-SMA* alpha-smooth muscle actin, *SDF-1* stromal cell-derived factor 1, *PI3K* Phosphoinositide 3-kinases, *AKT* Protein kinase B, *SOCS* Suppress cytokine signaling, *STAT3* Signal transducer and activator of transcription 3

### Exosomes promote angiogenesis in the pre-metastatic niche

To ensure adequate blood supply, VEGFR1^+^ hematopoietic progenitor cells initiate angiogenesis within the pre-metastatic niche [9]. Additionally, local endothelial progenitor cells promote angiogenesis via VEGF signaling [52]. Aside from blood vessel growth, the proangiogenic microenvironment increases vascular permeability towards the pre-metastatic niche for multiple cell types, including VEGFR1+ hematopoietic progenitor cells, immune cells, stromal cells, and the homing of tumor cells [52]. This antecedent angiogenesis makes the pre-metastatic niche capable of meeting the nutrient requirements for subsequent rapid metastatic growth. Ovarian cancer exosomes have recently gained notoriety for promoting angiogenesis (Fig. 2b). One such study demonstrated their ability to enhance viability and migration of human umbilical vein endothelial cells. At the molecular level, proteomics have revealed activating transcription factor 2 (ATF2) and metastasis-associated protein 1 (MTA1) housed within ovarian cancer exosomes to upregulate angiogenesis [58]. CD147 is a tumorigenic membrane-bound molecule expressed in cancer cells which regulates matrix metalloproteinase expression in peritumoral stromal cells. CD147-positive exosomes released by ovarian tumors promote angiogenesis in human umbilical vein endothelial cells as well [59]. In a uniquely VEGF-independent manner, soluble E-cadherin (sE-cad) harboring exosomes are present within the ascites of ovarian cancer patients and are robust activators of angiogenesis. Mechanistically, sE-cad-positive exosomes bind with VE-cadherin on endothelial cells, prompting a signaling cascade that ultimately activates β-catenin and NF-κB; this stimulates endothelial cell migration and overall vascular permeability [60]. Despite their small size, a growing body of research is supporting the role of ovarian cancer derived exosomes in cultivating an angiogenic tumor niche for widespread peritoneal metastasis.

### Exosomes in stromal remodeling

The survival of cancer cells that metastasize from primary tumors to secondary sites depends upon the stroma microenvironment. Tumor derived exosomes assist in this process by educating and remodeling stromal cells in the metastatic site to support tumor cell viability and metastatic dissemination. Functionally, they reprogram stromal cells in the pre-metastatic niche including cancer-associated fibroblasts (CAFs) and pericytes. Exosomes also interact with tumor-associated macrophages (TAM) in the metastatic microenvironment, which are responsible for tumor growth, invasion, angiogenesis, and overall metastasis.

### Exosomes convert fibroblasts to CAFs

CAFs are unique, reprogrammed stromal cells with roles in cancer initiation, extracellular matrix remodeling, progression, pre-metastatic niche formation, and metastasis [61]. They secrete a tumorigenic cytokine milieu of TGF-β, stromal cell-derived factor-1α (SDF-1α), S100A4, fibronectin, and matrix metalloproteinases in the local stromal cell microenvironment [62, 63]. While these signaling molecules have unique and specific processes in forming the tumor microenvironment, they all contribute to stromal remodeling within the pre-metastatic niche [62, 64]. In a study where the miRNA profile between normal fibroblasts and ovarian tumor-adjacent fibroblasts were compared, ovarian tumor-adjacent fibroblasts consistently showed miR-31 and miR-214 downregulation with miR-155 upregulation. These researchers demonstrated the significance of this miRNA signature by transfecting miR-31 and miR-214 mimics or miR-155 inhibitors into normal fibroblasts, which caused them to convert to CAFs [65]. This showed ovarian cancer derived exosomes alone are sufficient to induce the phenotypic and functional changes in normal stromal fibroblasts to pathogenic CAFs [66] (Fig. 2c). Similar work has supported the role of exosomes in transitioning normal stroma to CAFs. Ovarian cancer is able to transition adipose derived mesenchymal stem cells to CAFs by overexpressing alpha-smooth muscle actin (α-SMA), SDF-1 and TGF-β [67]. Reprogramming of normal stroma to cancerous stroma can therefore be mediated by cytokine signaling from exosomes prior to secondary metastatic growth.

### Exosomes induce macrophage polarization

Macrophages are multifunctional antigen presenting cells classically categorized into two polarized phenotypes: pro-inflammatory (M1) and anti-inflammatory (M2) [68]. Tumor associate-macrophages (TAMs) are of the M2 subtype and permeate malignant tissues [69]. Within the tumor microenvironment, TAMs secrete IL-4, IL-5, and IL-6, which promote angiogenesis, matrix remodeling, and immune system suppression [70]. They also contribute to the pre-metastatic niche by secreting TGF-β, SDF-1, and VEGF via the STAT3 signaling cascade [71]. In ovarian cancer, hypoxia-inducible factors (HIFs) induce the release of exosomes enriched with various miRNAs, including miR-21–3p, miR-125 b-5p, and miR-181 d-5p. When these exosomes are phagocytosed by undifferentiated macrophages, they undergo M2 polarization via the suppressor of cytokine signaling (SOCS)4/5/ STAT3 pathway [41] (Fig. 2d). One recent study showed ovarian cancer cells co-cultured with macrophages are only capable of transferring their oncogenic miR-1246 via exosomes to M2 macrophages and not to M1 macrophages. As a follow up, a combination miR-1246 inhibitor and chemotherapy regimen significantly reduced tumor burden in vivo [72]. These emerging works have supported the role of directing the M2 subtype at multiple stages of tumorigenesis and pre-metastatic niche formation.

### Exosome-educated cells in the pre-metastatic niche promote metastasis

**Fig. 3:**
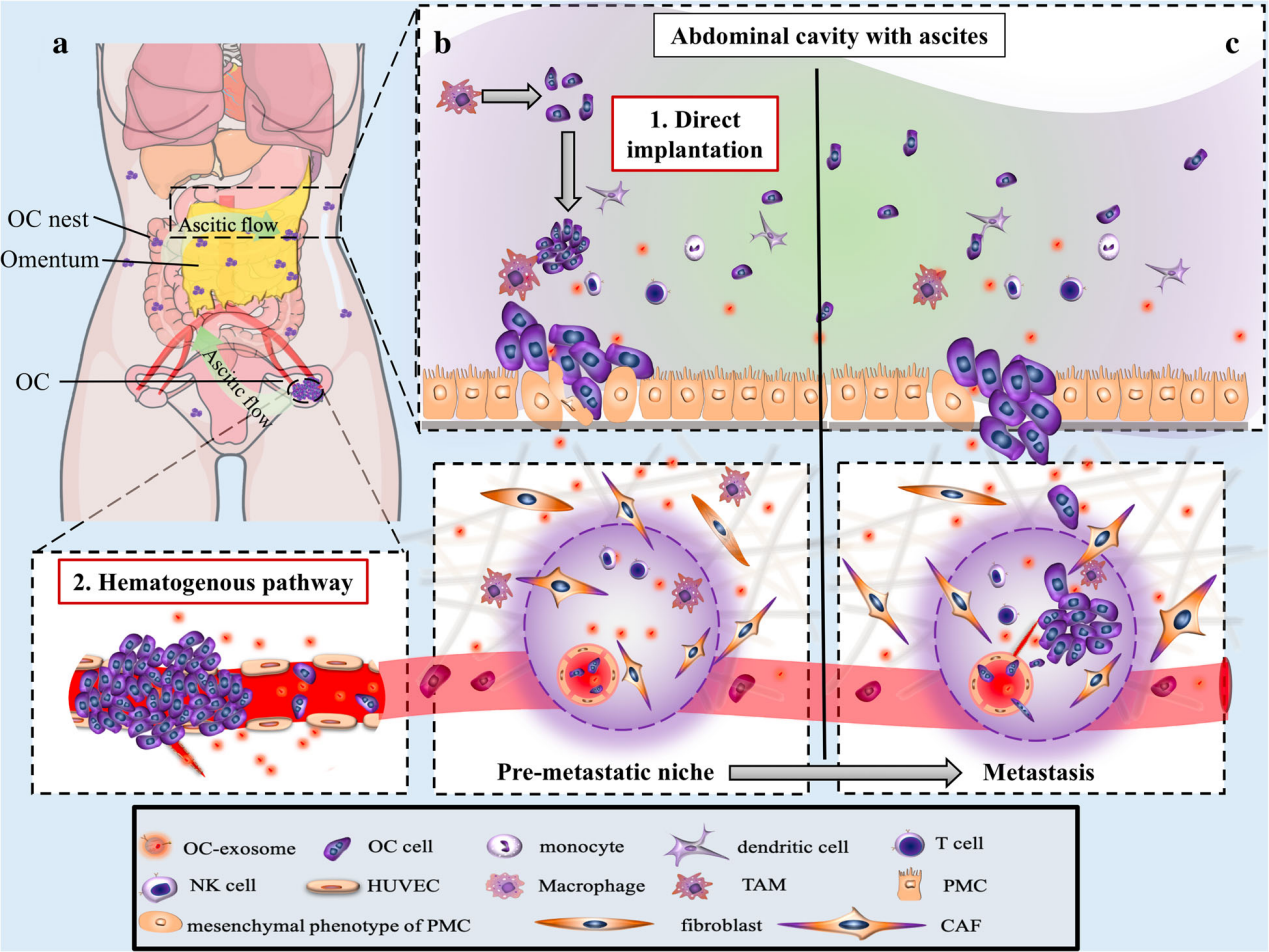
Two main methods of ovarian cancer metastasis within the peritoneal cavity, direct implantation and hematogenous spread. **a** Distribution of ovarian cancer nests in the peritoneal cavity. **b** Mechanisms of ovarian cancer-exosome establishment of the pre-metastatic niche before metastasis. **c** After the formation of pre-metastatic niche, tumor cells home to the metastatic site then colonize and support vessel budding. OC: ovarian cancer; NK: natural killer; HUVECs: Human umbilical vein endothelial cells; TAM: tumor-associated macrophages; PMC: Peritoneal mesothelial cell; CAF: cancer-associated fibroblasts.

Ovarian cancer exosomes directly promote circulating tumor cell homing, colonization, and outgrowth within the premetastatic niche while suppressing the host anti-tumor immune response. They also enable tumor cell proliferation and invasion by encouraging host cell transformation to TAM and CAF phenotypes (Table 3). Experimental evidence has shown exosome-educated TAMs to enhance ovarian cancer proliferation and migration in vitro and with in vivo mouse models [41]. This occurs as a result of the cytokine profile of TAMs, whereby they secrete large amounts of EGF which activate EGFR in peripheral ovarian cancer cells. This EGF/EGFR signaling cascade upregulates vascular endothelial growth factor-C (VEGF-C), which in turn upregulates integrin and intercellular adhesion molecule (ICAM-1). This induces proliferation, migration, adhesion, spheroid formation, and peritoneal implantation of ovarian cancer cells [73] (Fig. 3b). In the pre-metastatic niche, the tumor derived exosomes convert local fibroblasts into CAFs, which support tumorigenesis through their own exosome secretion. When ovarian cancer cells ingest TGFβ1-enriched CAF-exosomes, they upregulate TGFβ1 expression and become more adept at migration and invasion via a SMAD signaling cascade [74]. Aside from this function, CAFs also enhance basement membrane permeability allowing tumor cells to better invade the local uninvolved stroma [75].

There is a unique interplay between chemotherapeutic and exosome function. Intriguingly, it was recently shown that breast cancer exosomes have enhanced pre-metastatic niche forming ability in the lung after a chemotherapy application [76]. In another study, researchers showed the first-line chemotherapeutic cisplatin to increase IL-6-producing myofibroblastic CAFs in ovarian cancer patients by activating the NFκB signaling pathway [77]. Furthermore, they demonstrated pretreatment with the well-known diabetes drug metformin arrested stromal NFκB/IL6 activation and lessened the chemoresistance response in ovarian cancer cells [77]. Metastatic and drug-resistant, recurrent ovarian cancer produces significantly higher IL-6 compared to matched primary tumors. When elevated, IL-6 within the ascites of ovarian cancer patients correlates with poor outcomes [78–81]. Overall, current works are investigating IL-6 receptor antagonists, classically used to treat rheumatoid arthritis, for their potential anti-cancer effects [82].

### Exosomes breach the barriers to tumor invasion within the pre-metastatic niche

Approximately 70% of ovarian cancer patients have peritoneal metastasis at the time of surgery [83]. Typically, nests of tumor cells involve the peritoneum, omentum, mesentery, diaphragm, and surface of abdominopelvic viscera (Fig. 3a). These niduses of metastasis result from transport within the ascites; however, cases of hematogenous metastasis to the omentum also occur [84] (Fig. 3a). Histologically, both the omentum and peritoneum are loose collagen networks with vascularized connective tissue, scattered fibroblasts, adipocytes, and macrophages. They are covered by a single layer of microvilli-rich mesothelial cells and a thin basement membrane [85](Fig. 3b), which the circulating ovarian cancer cells must breach. The method of metastatic transport determines the barriers to invasion. In the hematogenous pathway, tumor cells penetrate surface endothelium, and, in the ascites pathway, they infiltrate the mesothelium (Fig. 3c).

Ovarian cancer exosomes advance angiogenesis by inducing vascular endothelial cell migration, which facilitates tumor cell homing to the pre-metastatic niche [58–60]. The notable absence of mesothelium in peritoneal viscera with metastatic growth suggests these cells are cleared during tumorigenesis, and possibly prior to large-scale metastatic invasion [86–88]. Mechanistically, ovarian cancer cells release exosomes with specific miRNAs (e.g miR-21 and miR-29a) into the ascites that remodel the mesothelial cell layer for enhanced peritoneal penetration [42] (Fig. 2e). Serum miR-99a-5p is significantly elevated in ovarian cancer patients and promotes cell invasion by affecting human peritoneal mesothelial cells (HPMCs) via fibronectin and vitronectin upregulation [43]. CD44 is overexpressed in the peritoneal mesothelial cells of ovarian cancer patients with omental metastasis. This occurs via an exosome conditioned pathway, whereby ovarian cancer exosomes transfer CD44 to peritoneal mesothelium causing its physical barrier to be cleared [37]. MMP1 expression is a negative factor of ovarian cancer prognosis. The mRNA of these proteases are horizontally transferred from ovarian cancer exosomes to peritoneal mesothelium, causing apoptosis of the peritoneal mesothelium through self-destruction [44].

### Exosomes as pre-metastatic niche biomarkers and therapeutic applications

Early detection of the pre-metastatic niche prior to metastasis remains an important goal within the field of gynecologic oncology, especially given the substantially worse outcomes associated with late stage diagnosis. Therefore, new techniques have sought to detect hyperpermeable, hypoxic, and inflammatory areas in addition to areas with altered extracellular matrix profiles characteristic of the metastatic niche. However, many of these techniques have low accuracy and are not yet suitable for clinical application [89–91]. Exosomes possess several unique advantages as biomarkers of pre-metastatic niche formation, as they are extremely stable, abundant, and tumor-specific. They are, therefore, promising biomarkers within the blood or ascites that warrant serious investigation in this deadly cancer, especially given their successes in other cancers [92]. For example, exosomes derived from pancreatic tumors bearing migration inhibitory factor (MIF) are selectively ingested by Kupffer cells within the liver, and therefore serve as important initiating factors in hepatic pre-metastatic niche formation [93]. Similarly, exosomal levels of the melanoma-specific protein tyrosinase-related protein 2 (TYRP2) has gained interest as a predictor of metastasis in melanoma [94].

Liquid biopsy is a technique where circulating tumor cells, cell-free nucleic acids, and tumor-derived exosomes can be analyzed from body fluids such as blood or ascites. The first exosome-based cancer diagnostic product was introduced to market within the United States on January 21, 2016 [95]. This technique is especially applicable to ovarian cancer diagnostics, as exosomes can be readily detected from ascites in a relatively non-invasive manner [96]. The most advanced biosensors in liquid biopsy detect cancer-derived exosomes via highly specific target selection, biologic antigen sensing, and signal transduction techniques [97]. As to target selection, levels of exosomal miR-200b and miR-200c are associated with poor outcomes in ovarian cancer and significantly correlate with the ovarian tumor marker CA-125 [98]. Cancer-related antigens are prominent on the surface of exosomes and amenable to highly sensitive cancer cell detection. Examples include exosomes enriched with VEGF-A, semaphorin-3A, and TGF-beta in glioma (GBMs), HIF1-α in nasopharyngeal carcinoma, and MT1 MMP in fibrosarcoma and melanoma [22]. These proteins associated with exosomes are unique to their cancer of origin, and therefore promising targets of liquid biopsy detection especially for patients with an unknown cancer subtype. To this effect, various malignancies have been studied including melanoma, nasopharyngeal carcinoma, breast cancer, colorectal cancer, and ovarian cancer [97]. Very recently, a microfluidic chip-based liquid biopsy was able to isolate exosomes with detailed protein and signaling pathway profiles in ovarian cancer [99].

Therapeutic exosome-based strategies are also emerging, and function by exploiting the homing effect of exosomes on primary cancer cells [100]. A pre-metastatic niche mimic was generated by embedding ovarian cancer-exosomes into engineered biomaterials and implanted within the abdominal cavity of a murine model. This artificial pre-metastatic niche effectively recruited and trapped free ovarian cancer cells from the ascites, thus preventing these cells from homing towards normal pelvic viscera. Survival was substantially increased [101]. Another emerging technology utilizes cell-targeting aptamer-modified extracellular vesicles with exosomes embedded in black phosphorus. While the aptamer directs the bioinspired extracellular vesicles towards targeted cells, the black phosphorous derived inorganic phosphate facilitates cell biomineralization [102]. This targeted technology has potential for elimination of the pre-metastatic niche and is a novel area of future chemotherapeutic research.

Tumor-derived exosomes undergo homing to preferred organ and cell-specific sites when preparing the pre-metastatic niche. Exosomal proteomics have revealed expression patterns of integrins to play a major role in this process. While the exosomal integrins α_6_β_4_ and α_6_β_1_ promote lung metastasis, α_v_β_5_ is linked to liver metastasis. Therapeutically, targeting integrins α6β4 and αvβ5 reduces exosome uptake and lung and liver metastasis, respectively [103]. These exosomal integrins are an additional predictor of pre-metastatic niche formation in liquid biopsy, and especially valuable for cancers with high rates of metastasis. Overall, because different cancers exhibit distinct mechanisms and exosome profiles in their respective pre-metastatic niches [93, 103, 104], it is likely that tumor specific biomarkers and therapeutic strategies will have acceptable specificity. In addition, given the pre-metastatic biomarker profile housed within ovarian cancer exosomes and their roles in pre-metastatic niche formation, recent advances in liquid biopsy diagnostics have made exosomes a promising new area of early screening and detection (Table 3).

## Conclusions and perspectives

Ovarian cancer exosomes promote pre-metastatic niche formation via immunosuppression, angiogenesis, stromal cell remodeling, and oncogenic reprogramming (Fig. 2). Since the pre-metastatic niche gained critical attention in 2005 [9], significant progress has been made in understanding the contribution of exosomes in conditioning the pre-metastatic niche for subsequent rapid metastatic growth. However, the roles of cell-shed exosomes have only recently been appreciated for their substantial effects on shaping the tumor microenvironment. In the preclinical work to date, exosomes in animal studies have been isolated and purified in vitro. Emerging technologies such as liquid biopsy will likely further characterize their tumorigenic effects in vivo, and may help to fully reveal the clinical significance of these pro-metastatic factors in ovarian cancer.

## Data Availability

Not applicable.

## Abbreviations

ADSCs: Adipose tissue-derived mesenchymal stem cells
AKT: Protein kinase B
ALIX/PDCD6IP: programmed cell death 6-interacting protein
ARF6: ADP-ribosylation factor 6
ATF2: Activating transcription factor 2
COIA1: Collagen alpha-1 (XVIII) chain
DCs: Dendritic cells
ECs: Endothelial cells
EOC: Epithelial ovarian cancer
EOC: Epithelial ovarian cancer
EPHA2: Ephrin type-A receptor 2
ESCRT: Endosomal sorting complex required for transport
FasL: Fas ligand
GD 3: Ganglioside 3
GNA12: Guanine nucleotide-binding protein subunit alpha-12
HEK293: Human embryonic kidney
HIF: Hypoxia-inducible factor
HPMC: Human peritoneal mesothelial cells
HPMCs: Human peritoneal mesothelial cells
HUVECs: Human umbilical vein endothelial cells
HUVECs: Human umbilical vein endothelial cells
ILV: Intraluminal vesicle
LIN28: Lin-28 homolog A
LPA: Lysophosphatidic acid
MMP: Matrix metallopeptidase
MSCs: Mesenchymal stem cells
MTA1: Metastasis-associated protein 1
MVEs: Multivesicular endosomes
NK: Natural killer
OC: Ovarian cancer
PBMCs: Peripheral blood lymphocytes
PI3K: Phosphoinositide 3-kinases
PS: Phosphatidylserine
RISCs: Coiled-Coil Containing Protein Kinase
RNA: Induced silencing complexes
ROCK: Rho-Associated
SDF-1: Stromal cell-derived factor 1
sE-cad: Soluble E-cadherin
SOCS: Suppress cytokine signaling
STAT3: Signal transducer and activator of transcription 3
TAMs: Tumor-associated macrophages
TCR: T-cell receptor
TLR: Toll-like receptor
TSG101: Tumor susceptibility gene 101 protein
VPS4: Vacuolar protein sorting-associated protein
α-SMA: Alpha-smooth muscle actin
